# Effects of Different Hypothermia on the Results of Cardiopulmonary Resuscitation in a Cardiac Arrest Rat Model

**DOI:** 10.1155/2022/2005616

**Published:** 2022-04-04

**Authors:** Shaohua Xu, Hui Miao, Liming Gong, Lijie Feng, Xuliang Hou, Manhong Zhou, Hong Shen, Wei Chen

**Affiliations:** ^1^Nankai University School of Medicine, Tianjin, China; ^2^The 1st Medical Center of Chinese PLA General Hospital, Beijing, China; ^3^The 3rd Medical Center of Chinese PLA General Hospital, Beijing, China; ^4^Affiliated Hospital of Zunyi Medical University, Guizhou, China; ^5^Hainan Hospital of Chinese PLA General Hospital, Hainan, China

## Abstract

**Objective:**

To investigate the optimal temperature of hypothermia treatment in rats with cardiac arrest caused by ventricular fibrillation (VF) after the return of spontaneous circulation (ROSC).

**Methods:**

A total of forty-eight male Sprague-Dawley rats were induced by VF through the guidewire with a maximum of 5 mA current and untreated for 8 min. Cardiopulmonary resuscitation (CPR) was performed for 8 min followed by defibrillation (DF). Resuscitated rats were then randomized into the normothermia (37°C) group, milder (35°C) group, mild (33°C) group, or moderate (28°C) group. Hypothermia was immediately induced with surface cooling. The target temperature was maintained for 4 h before rewarming to 37 ± 0.5°C. Moreover, at the end of the 4 h, a rat in each group was randomly selected to be sacrificed for the cerebral cortex electron microscopy observation (*n* = 1). The other resuscitated animals were observed for up to 72 h after ROSC (*n* = 7). Left ventricular ejection fraction (LVEF) and left ventricular end diastolic volume (LVEDV) were measured. Survival time, survival rate, and neurological deficit score (NDS) were recorded for 72 h.

**Results:**

During hypothermia, higher LVEF was observed in the hypothermia groups when compared with normothermia group (35°C vs. 37°C, *p* < 0.05, 33°C and 28°C vs. 37°C, *p* < 0.01). Among the hypothermia groups, LVEF was higher in the 28°C group than that of 35°C (*p* < 0.05). However, both the heart rate (HR) (*p* < 0.01) and LVEDV (28°C vs. 35°C, *p* < 0.01, 28°C vs. 37°C and 33°C, *p* < 0.05) were lowest in the 28°C group when compared with the other groups. There were no significant differences of LVEF and LVEDV between the group 35°C and 33°C (*p* > 0.05). After rewarming, the LVEF of 35°C group was higher than that of group 37°C, 33°C, and 28°C (35°C vs. 37°C and 28°C, *p* < 0.01, 35°C vs. 33°C, *p* < 0.05). Group 35°C and 33°C resulted in longer survival (*p* < 0.01), higher survival rate (*p* < 0.01), and lower NDS (35°C vs. 37°C and 28°C, *p* < 0.01, 33°C vs. 37°C and 28°C, *p* < 0.05) compared with the group 37°C and 28°C. The extent of damage to cerebral cortex cells in group of 35°C and 33°C was lighter than that in group of 37°C and 28°C. The 35°C group spent less time in the process of cooling and rewarming than the group 33°C and 28°C (*p* < 0.01).

**Conclusions:**

An almost equal protective effect of milder hypothermia (35°C) and mild hypothermia (33°C) in cardiac arrest (CA) rats was achieved with more predominant effect than moderate hypothermia (28°C) and normothermia (37°C). More importantly, shorter time spent in cooling and rewarming was required in the 35°C group, indicating its potential clinical application. These findings support the possible use of milder hypothermia (35°C) as a therapeutic agent for postresuscitation.

## 1. Introduction

Currently, cardiac arrest (CA) has a high mortality rate worldwide, and only about 10.3-12% of patients survive to hospital discharge or 30 days [1, 2]. Therefore, in order to improve the survival rate of CA patients, the number of researchers in return of spontaneous circulation (ROSC) has increased, and substantial progress has been made. However, many interventions improve ROSC without an achievement of long-term survival. In different countries and regions, about 25% of out-hospital cardiac arrest (OHCA) patients can ROSC when they arrive at the hospital [1], while only about 25-50% of all ROSC patients can survive to discharge [3].

Studies have found that prolonged cardiopulmonary resuscitation (CPR) may cause irreversible brain injury [4, 5] which is one of main causes of in-hospital death of CA patients caused by ischemia and hypoxia [3]. In the postresuscitation settings, therapeutic hypothermia has been proven to be one of the effective ways in increasing survival rates. Previous studies demonstrated that hypothermia reduced the metabolic rate and reperfusion injury [6–9]. Studies by Grand et al. also found [10] that target temperature management (TTM) for CA patients and maintain body temperature below 36°C can reduce systemic oxygen consumption, and systemic oxygen consumption decreases by 19 ml O_2_ for every 1°C drop in body temperature. TTM through hypothermia and prevention of hyperthermia has been shown to improve survival and neurological results after cardiac arrest [11, 12]. Studies have found that CA patients with TTM treatment have less brain nerve damage at 90 days and higher survival rate than without [13].

The American Heart Association (AHA) recommends that CA patients maintain their body temperature between 32°C and 36°C [14]. The latest cardiopulmonary resuscitation guidelines also recommend cooling the treatment body temperature after ROSC down to 32-36°C for 24 h.

However, previous guidelines for cardiopulmonary resuscitation recommend that the TTM after resuscitation maintenance at 32-34°C, and some studies have pointed out that as body temperature drops, systemic oxygen consumption also gradually decreases. Nevertheless, in recent years, studies have found that patients receiving ROSC with TTM at 36°C have no significant difference in long-term cognitive function and mortality with that at 33°C or 34°C [15–17]. Moreover, patients treated with a target temperature of 34-36°C have fewer acute complications [18].

It is easier to achieve the target body temperature of 36°C compared than lowering and maintaining the body temperature at 32-34°C. However, some studies have pointed out that when the body temperature is stable at 36°C, CA patients have a lower compliance with the target body temperature, a higher fever rate, and a trend of clinical deterioration in the patient's prognosis [19]. Another study found that less neurological deterioration was observed at 33°C than 36°C, although there was no significant difference in mortality of both patients [20]. Moreover, it is difficult to maintain the body temperature at 36°C without fluctuations in clinical practical applications.

Although the current cardiopulmonary resuscitation guidelines and AHA have clearly recommended that the target body temperature after ROSC being controlled at 32-36°C, the optimal cooling body temperature has not yet been determined within this temperature range. We hypothesize that milder hypothermia (35°C) has the same protective effect on the outcome after resuscitation compared with mild hypothermia (33°C) and produces significantly better outcomes compared with normal temperature and moderate hypothermia (28°C).

## 2. Materials and Methods

### 2.1. Animals

Healthy adult male clean-grade Sprague-Dawley rats, purchased from Changsha Tianqin Biotechnology Co., Ltd., license number: SCXK (Xiang) 2019-0014, were raised in separate cages, room temperature (22 ± 1°C), 12 h/12 h day and night cycle, free eating, and drinking. All rats were adaptively reared for 1 week before the experiment. The experiment process complies with the state and the school's management regulations and ethical requirements for experimental animals.

### 2.2. Animal Preparation

Studies were performed in 48 male Sprague-Dawley rats, weighing between 450 and 500 g. Animals were fasted overnight except for free access to water and anesthetized by intraperitoneal injection of pentobarbital (45 mg/kg). Anesthesia was maintained with additional doses of pentobarbital (5 mg/kg), which was administered when required to maintain anesthesia. The trachea was exposed by pulling the tongue to one side with the tissue forceps and cannulated with a catheter (14-gauge, Calvin Biotechnology Co., Ltd., China). A conventional lead II EKG was continuously monitored. A 23-gauge polyethylene catheter (PE-50, Becton-Dickinson, USA) was advanced through the left external jugular vein through the superior vena cava into the right atrium for measurement of right atrial pressures. A 3-F PE catheter (C-PMS-301J, Cook Medical, USA) was advanced through the right external jugular vein into the right atrium. A guidewire supplied with the catheter was then advanced through the catheter into the right ventricle to induce ventricular fibrillation (VF). An additional PE-50 catheter was advanced through the left femoral artery into the thoracic aorta for measurement of mean aortic pressure (MAP). An electronic thermometer probe provided by monitoring system was inserted into the rectum for core temperature measurement. All catheters were flushed intermittently with saline containing 2.5 IU/ml of crystalline bovine heparin.

### 2.3. Experimental Procedures

Baseline (BL) measurements were observed 10 min before the induction of VF. Animals were mechanically ventilated with a tidal volume of 0.65 ml/100 g body weight and a frequency of 100 breaths/min. The inspired O_2_ fraction (FiO_2_) was maintained at 0.21. VF was induced to the right ventricular endocardium through the guidewire with a maximum of 5 mA current. Mechanical ventilation was stopped at the same time. Precordial chest compression was started with a preprepared mechanical chest compressor providing 200 compressions/min after 8 min of untreated VF. The depth of compression was adjusted to maintain a coronary perfusion pressure (CPP) of 22 ± 2 mmHg. Mechanical ventilation was started with a tidal volume of 0.65 ml/100 g body weight and an FiO_2_ of 1.0. defibrillation (DF) was attempted with up to three two-joule counter shocks after 8 min CPR. CPR was continued for 30 sec before the next DF if spontaneous circulation was not restored. The protocol was continued until successful resuscitation or the sequence was repeated three cycles. Animals were regarded as successfully resuscitated if ROSC was achieved in minimum 5 min with a mean aortic pressure of more than 50 mmHg [21]. Resuscitated animals were randomized into four groups according to the different temperature settings. Group I: normothermia group (37°C group); group II: milder hypothermia group (35°C group); group III: mild hypothermia group (33°C group); group IV: moderate hypothermia group (28°C group). Then, hypothermia was induced immediately. The target temperature should be reached in 4 min, 9 min, or 24 min with the aid of ice packs and electrical fans according to the different groups. Once the target temperature was reached, animals were maintained with cooling blanket for 4 h before rewarming. One rat in each group was randomly selected to be sacrificed at the end of the 4 h, and the cerebral cortex was taken for electron microscopy experiments. The remaining 7 rats in each group were rewarmed with a rate of 1°C in the 1^st^ h and 2°C/h until the body temperature recovered to 37 ± 0.5°C. The FiO_2_ was reduced from 1.0 to 0.5 at 30 min after resuscitation and further reduced to 0.21 at 1 h. All catheters, including the endotracheal tube, were removed after the animals had recovered from anesthesia. After rewarming, the rats were back to their own cages equipped with a heated pet mat to maintain the body temperature at 37°C for 12 h and were closely observed up to 72 h after ROSC.

### 2.4. Measurements

Heart rate (HR), aortic and right atrial pressures, electrocardiogram, and rectal temperature were continuously measured and recorded on a multifunction monitoring system (GE Solar 8000, GE Medical Systems Information Technology, USA). CPP was calculated as the difference between decompression diastolic aortic and time-coincident right atrial pressure. Left ventricular ejection fraction (LVEF) and left ventricular end diastolic volume (LVEDV) were measured by echocardiography, at BL, during hypothermia and after rewarming. Rats' cerebral cortex was taken at the end of hypothermia for electron microscopy (HT7800, HITACHI, Japan) experiments.

Neurological deficit score (NDS) developed by Hendrickx HH [22, 23] was measured for evaluating neurological recovery at 24 h intervals for a total of 72 h. Duration of survival was also recorded up to 72 h after ROSC.

### 2.5. Statistical Analysis

All data were presented as mean ± standard deviation (SD). Normal distribution was confirmed with the Kolmogorov-Smirnov test. For measurements among groups that conform to a normal distribution, analysis of variance (ANOVA) and Scheffé's multiple-comparison techniques were employed. And for nonnormally distributed between-group measurements, the Mann–Whitney *U* method was used for comparison. Comparisons between time-based measurements within each group were performed with repeated-measurement analysis of variance. The survival rate was analyzed with Fisher's exact test. A value of *p* < 0.05 was regarded as significant.

## 3. Results

### 3.1. Animal Features

Of 48 rats screened for the protocol, three were excluded during animal preparation because of anesthesia and bleeding. An additional 13 rats were excluded because of nonresuscitated. In this fashion, 32 rats were randomized into four experimental groups. BL measurements including animal body weight, rectal temperature, HR, LVEF, and LVEDV did not differ significantly among the four groups (Table 1). Before rewarming, one rat was used in the cerebral cortex electron microscope experiment in each group. Therefore, in order to maintain the consistency of data analysis, in the present study, in addition to the basic situation analysis before cooling treatment, 8 rats in each group were used, while the 7 rats of each group were used for statistical analysis.

### 3.2. Temperature

**Figure 1:**
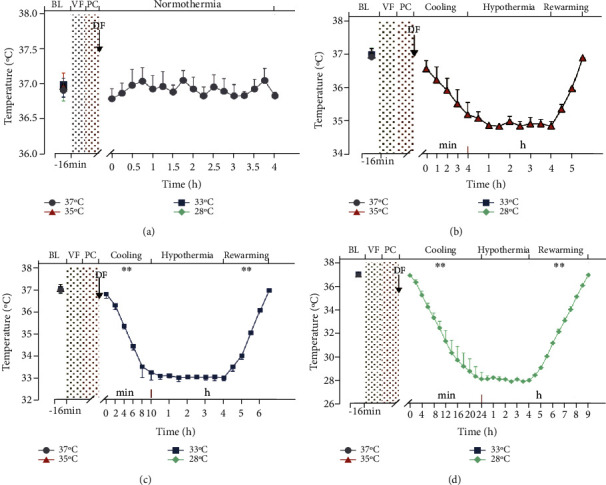
Temperature control of rats in four groups. Rats in 37°C group (a) maintain normothermia. 35°C group (b), 33°C group (c), and 28°C group (d) reached the target temperature within 4 min, 9 min, and 24 min. After reaching the target temperature, it will be maintained for 4 h before rewarming. Significantly more time was used for cooling and rewarming process in group 33°C and 28°C than 35°C. ∗∗*p* < 0.01 compared to the 35°C group. PC: precordial compression; *n* = 7.

Core temperature was strictly controlled. The temperature in all groups reached to the target set in protocol and was rewarmed at the same rate after hypothermia therapy (Figure 1). The 35°C group spent significantly less time in the process of cooling and rewarming than the group 33°C and 28°C (Figure 1).

### 3.3. Hypothermia

**Figure 2:**
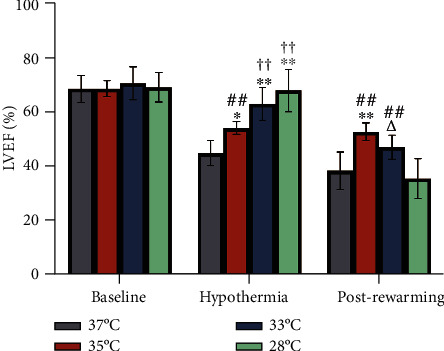
LVEF during hypothermia and postrewarming. During the hypothermia treatment, the LVEF of group 35°C, 33°C, and 28°C was higher than that of group 37°C. And in this period, the LVEF of 35°C group was significantly lower than that of 28°C group. However, in the postrewarming, the LVEF of 35°C group was significantly higher than that of group 37°C, 33°C, and 28°C. ∗*p* < 0.05, ∗∗*p* < 0.01 compared to the 37°C group; ^##^*p* < 0.01 compared to the 28°C group; ^∆^*p* < 0.05 compared to 35°C group; ^††^*p* < 0.01 compared to postrewarming within the same group. *n* = 7.

**Figure 3:**
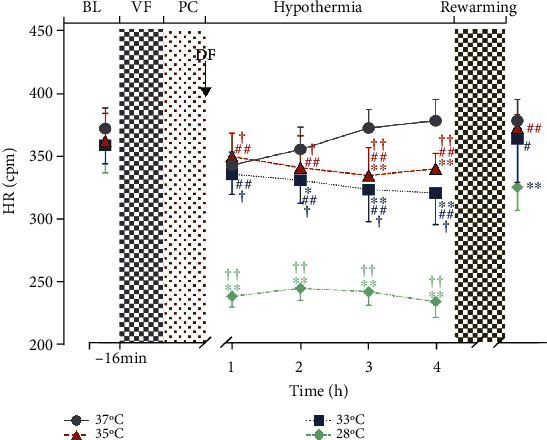
HR during hypothermia and rewarming. During the hypothermia treatment, the HR of the 28°C group decreased significantly, and the group 35°C and 33°C were significantly higher than that of the 28°C group. The group 33°C and 28°C were significantly higher than that of the 37°C group. In addition, the group 35°C, 33°C, and 28°C were lower than postrewarming within the same group. No significant difference was observed between the group 35°C and 33°C (*p* > 0.05). ∗*p* < 0.05, ∗∗*p* < 0.01 compared to the 37°C group; ^#^*p* < 0.05, ^##^*p* < 0.01 compared to the 28°C group; ^†^*p* < 0.05, ^††^*p* < 0.01 compared to postrewarming within the same group. *n* = 7.

**Figure 4:**
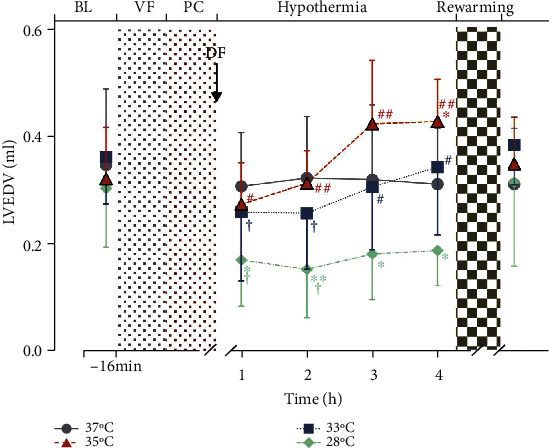
Left ventricular end diastolic volume during hypothermia and rewarming. During the hypothermia treatment, the 28°C group decreased significantly, and the 35°C group was significantly higher than that of the 28°C group, and the 33°C group was also higher than the 28°C group at the 3rd h of the hypothermia treatment. In addition, the group 33°C and 28°C within 2 h of starting hypothermia treatment were lower than postrewarming within the same group. No significant difference was observed between the group 35°C and 33°C (*p* > 0.05). ∗*p* < 0.05, ∗∗*p* < 0.01 compared to the 37°C group; ^#^*p* < 0.05, ^##^*p* < 0.01 compared to the 28°C group; ^†^*p* < 0.05 compared to post-rewarming within the same group. *n* = 7.

**Figure 5:**
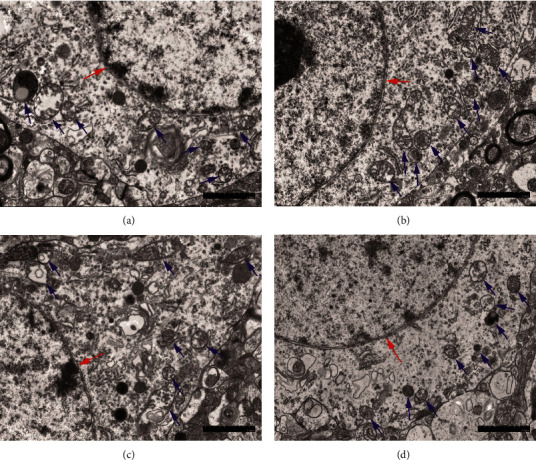
Scanning electron microscopy of cerebral cortex at different target temperatures at the 4th h of hypothermia. The cerebral cortex cells of rats in each group had damage to various extents. In the electron microscope picture, the nuclear membrane (red arrow) is blurred, the intracellular mitochondria (blue arrow) are swollen, and the arrangement of the cristae structure is obviously disordered, reduced, and disappeared, forming vacuoles. However, the damage of 35°C group (b) and 33°C group (c) is significantly lighter than that of 37°C group (a) and 28°C group (d). Bar = 2 *μ*m. *n* = 1.

Compared to the normothermia group, higher LVEF was observed in the hypothermia groups during hypothermia (Figure 2). Among the three groups, LVEF was higher in the 28°C group when compared with 35°C group (Figure 2). However, statistically significant reductions in HR (Figure 3) and LVEDV (Figure 4) were observed in 28°C group. Between the group 33°C and 35°C, however, there was no difference in LVEF, HR, and LVEDV (Figures 2–4). Electron microscopy of the cerebral cortex at the 4^th^ h after ROSC treatment with different body temperatures showed that the cerebral cortex was more damaged under 37°C or 28°C than the other two conditions where the nuclear membrane was blurred and broken; the intracellular mitochondria was swollen, the cristae structure arrangement was disordered, reduced, and disappeared; and vacuole was formed (Figure 5).

### 3.4. Postrewarming

After rewarming, a better LVEF was observed in both group 35°C and 33°C when compared with 28°C group, and greater LVEF in the 35°C group was observed when compared with the 33°C group (Figure 2). Interestingly, when compared in the same group, LVEF was not different significantly with the 35°C group after rewarming, and a significant decrease was observed in both group 33°C and 28°C (Figure 2). HR was increased in all hypothermia groups after rewarming (Figure 3).

### 3.5. Survival and NDS

**Figure 6:**
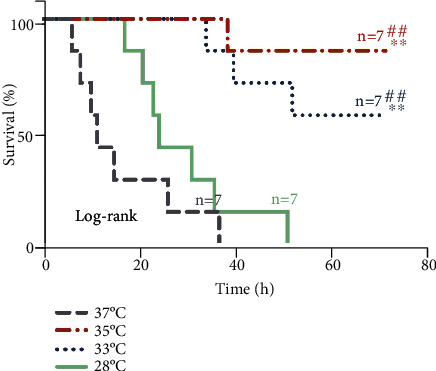
Survival analysis. All rats in the group 37°C, 35°C, 33°C, and 28°C after ROSC were observed for 72 h, and the survival curves were drawn. Kaplan-Meier survival analysis using log-rank test. The survival time and survival rate of group 35°C and 33°C were significantly higher than those of group 37°C and 28°C (*p* < 0.01). No significant difference of survival time and survival rate was observed between the group 35°C and 33°C (*p* > 0.05). ∗∗*p* < 0.01, ^##^*p* < 0.01. *n* = 7.

Duration of survival and survival rate was better in both group 33°C and 35°C when compared with the group 28°C and 37°C (Figure 6). This probably was associated with better NDS in both group 33°C and 35°C (Table 2). And no significant difference of duration of survival, survival rate, and NDS were observed between the group 33°C and 35°C (Figure 6, Table 2).

The neurological function of group 35°C and 33°C are better than that of group 37°C and 28°C. No significant difference of neurological function was observed between the group 35°C and 33°C and between the group 37°C and 28°C. ∗*p* < 0.05, ∗∗*p* < 0.01 compared to the 37°C group; ^#^*p* < 0.05, ^##^*p* < 0.01 compared to the 28°C group. Values are presented as mean ± SD. *n* = 7.

### 3.6. Summarization

**Figure 7:**
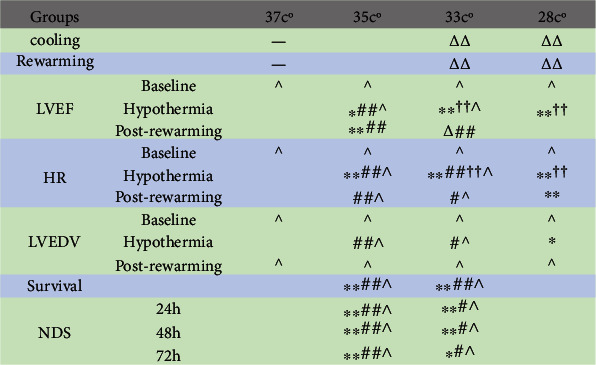
Summary of results. ∗*p* < 0.05, ∗∗*p* < 0.01 compared to the 37°C group; ^#^*p* < 0.05, ^##^*p* < 0.01 compared to the 28°C group; ^∆^*p* < 0.05, ^∆∆^*p* < 0.01 compared to 35°C group; ^††^*p* < 0.01 compared to postrewarming within the same group; ^: not significant between the different groups.

We summarized the effect of treatment at different temperature conditions after ROSC (Figure 7).

## 4. Discussion

In routine practice work, many patients with CA have achieved ROSC through treatment measures such as CPR, but eventually lead to adverse neurological outcomes due to ischemic and hypoxic brain damage. In the postresuscitation settings, only hypothermia has been approved to have neurological protection and increase survival chance. The current guidelines recommend cooling to 32°C to 36°C as one of the standard measurements during postresuscitation, but the optimal cooling temperature remains unknown. For this reason, we compare and analyze the treatment at different body temperatures after ROSC. The results showed that the hypothermia group had better LVEF than the normal body temperature group. The comparable survival time, survival rate, and neurological function were also observed in the group 35°C and 33°C, while there were no significant differences in survival rate, HR, myocardial function, NDS, and neurological function between the group 35°C and 33°C.

However, mild hypothermia (33°C) performed in clinical is suboptimally. The time needed to achieve the target temperature varies depending on the different cooling devices and methods [24–26]. With the conventional way of cooling (the rapid infusion cold saline and surface cooling with ice and/or cold packs) in the emergency setting, the cooling rate was as low as 0.5°C/h in human which may reduce the benefits in the hypothermia [27]. Mild hypothermia (33°C) was easier to overcooling to moderate hypothermia which resulted in more side effects of hypothermia [28, 29]. After rewarming from mild hypothermia (33°C), patients always experience rebound hyperthermia of >38°C which may exacerbate neurological damage [26]. In addition, mild hypothermia (33°C) impaired postresuscitation myocardial diastolic function by decreasing temperatures [30, 31]. However, a clinical study showed that hypothermia at 35°C achieved target temperature rapidly, and there is no need to treat shivering by anesthesia which will reduce the clinical work load. And there was no fever after rewarming from hypothermia [32].

As became increasingly problematic of mild hypothermia (33°C) has been found during the clinical practice, it was imperative to maximize the benefits target body temperature with the same protective effect and almost no side effects within the current recommended temperature range (32~36°C).

Postresuscitation myocardial dysfunction, including systolic and diastolic left ventricular function, is a common event in prolonged cardiac arrest and can have life-threatening consequences which also makes for the low survival rate after in- and out-of-hospital cardiac arrest [33–35]. In swine studies, LVEF decreased from 55% to 20%, and left ventricular end-diastolic pressure increased from 10 to 14 mmHg to 16 to 22 mmHg as early as 30 min after ROSC [35, 36].

Milder-induced hypothermia may be beneficial to the myocardium in a similar way that it is neuroprotective to the brain and reduce the mortality of post-CPR [37, 38]. There are the comparable pathological processes occurred in the heart and brain following cardiac arrest, including reperfusion and reoxygenation injury. Actually, previous studies demonstrated milder hypothermia (35°C), with only a small reduction (1.5-2.0°C) in temperature, benefits from the myocardium, such as reducing the myocardium infarct size and improving remodeling process as well [39–41].

Hypothermia increased inotropy to improve the systolic dysfunction [30, 42], at the expense of diastolic dysfunction [30, 31]. Previous studies demonstrated that LVEF increased at the temperature below 33°C, but diastolic dysfunction was noted [42, 43]. Diastolic cardiac function was impaired progressively by decreasing temperatures which might shorten the diastolic time interval, delay active relaxation, and decrease end-diastolic distensibility. Moderate hypothermia (28°C) leaded to worse diastolic function and worsened myocardial damage when compared with the other three groups [31, 44].

All these results were comparable to that of the present study, LVEF was significantly higher in the hypothermia groups when compared with normothermia group. However, among hypothermia groups, moderate hypothermia had better LVEF than milder hypothermia (35°C) but with lower LVEDV due to worsening end diastolic distensibility [30]. After rewarming, a significant lower LVEF was observed in the moderate hypothermia for the myocardial damage when compared with mild and milder hypothermia [44]. The present study indicated LVEF with hypothermia at lower temperature was more sensitive to the change in temperature. During hypothermia therapy, body temperature should be managed carefully in case of overcooling and unintentional rewarming. Hypothermia at lower temperature should be rewarmed more slowly and carefully.

Postcardiac arrest cerebral injury is a common cause of morbidity and mortality [33]. Hypothermia could reduce cerebral metabolic rate and protect against many of the processes associated with reperfusion and reoxygenation which cause neurological injury [6–8, 45]. Another study demonstrated that hypothermia treatment can inhibit the apoptosis of injured neuronal cells, but when the hypothermia is lower than 30°C, it may induce apoptosis in intact cells [46]. The ability of hypothermia to alleviate neuronal cell damage may be related to the increased expression of RNA-binding motif protein 3 (RBM3) and the promotion of neurogenesis [47]. Mild hypothermia (33°C) improved neurological outcome and reduced mortality among initially comatose survivors of out-hospital cardiac arrest [11, 12]. Simultaneously, milder hypothermia (35°C) has the neurological protective effect as well. Jahandiez et al. [48] found that the application of hypothermia during reperfusion can alleviate brain resuscitation injury by inhibiting the opening of permeability transition pore (PTP) and the activation of the protein kinase B (Akt) component of the prosurvival Reperfusion Injury Salvage Kinase (RISK) pathway. Animal studies have shown that cooling to 35°C had the effect to inhabit the ischemia/reperfusion injury, reduce apoptosis, and improve neurological scores and neuronal survival which had similar benefits to 33°C [49–51]. This study also confirmed that the DNS and level of brain injury in the group 33°C and 35°C were lower and lighter than that in the group 28°C and 37°C, which may also be one of the main reasons why the survival and survival rate of the first two groups of rats to be higher than that of the latter two groups. However, there was no significant difference in survival, survival rate, DNS, and level of brain injury between the two groups at 33°C and 35°C. This indicated that the milder hypothermia (35°C) exert the comparable protective effect with mild hypothermia (33°C) to survival rate and central nervous function protection in this model.

We observed several limitations in the study. First, coronary disease or other organic heart disease is not incorporated into the model that mostly complicates cardiac function during postresuscitation. Second, the rewarming rate is rapid in the present study compared with recommended range (0.25 to 0.5°C/h) in some studies, but there is still no unified optimal rewarming rate [52]. This maybe influence long-term survival in the study [53]. However, if we followed the recommended rewarming rate, it would take a long time to keep the animals in low temperature. A study reported that the neuroprotective effect of hypothermia treatment at 32°C or 34°C for 6 h is not as predominant as that of 4.5 h[54]. It indicated that duration of hypothermia affected survival rate, leading to the use of a rapid rewarming rate for the study. Although the rewarming rate was rapid in the present study, we still could find the different outcomes among the three hypothermia groups. Finally, because of the same rate of rewarming, the three hypothermia groups required different times to recover the normal body temperature. The postrewarming measurements were not taken at the same intervals after ROSC which may affect the results of postrewarming. However, we compared the postrewarming with the hypothermia within the group which help to assess the results of hypothermia.

## 5. Conclusions

An almost equal protective effect of milder hypothermia (35°C) and mild hypothermia (33°C) in cardiac arrest (CA) rats was achieved with more predominant effect than moderate hypothermia (28°C) and normothermia (37°C). More importantly, shorter time spent in cooling and rewarming was required in the 35°C group, indicating its potential clinical application. These findings support the possible use of milder hypothermia (35°C) as a therapeutic agent for postresuscitation.

## Figures and Tables

**Table 1:** BL before applying hypothermia.

Groups	Weight (*g*)	Temperature (°C)	HR (cpm)	LVEF (%)	LVEDV (ml)
37°C	479.3 ± 12.9	36.91 ± 0.16	370.13 ± 15.62	71.98 ± 5.79	0.35 ± 0.13
35°C	481.1 ± 12.3	36.97 ± 0.18	360.88 ± 20.39	68.77 ± 2.88	0.32 ± 0.1

33°C	478.6 ± 8.0	36.99 ± 0.16	357.5 ± 13.63	69.55 ± 5.06	0.36 ± 0.09
28°C	480.8 ± 12.0	36.99 ± 0.20	358.38 ± 20.09	71.06 ± 5.73	0.3 ± 0.1

Values are presented as mean ± SD. g: gram; cpm: counts per min. *n* = 8.

**Table 2:** NDS at different time points after ROSC.

Groups	24 h	48 h	72 h
37°C	467.9 ± 59.0	500.0 ± 0.0	500.0 ± 0.0
35°C	302.9 ± 53.1∗∗^##^	230.7 ± 163.5∗∗^##^	185.7 ± 217.5∗∗^##^

33°C	336.4 ± 57.6∗∗^#^	308.6 ± 155.7∗∗^#^	265.0 ± 221.4∗^#^
28°C	427.1 ± 74.6	489.3 ± 28.3	500.0 ± 0.0

## Data Availability

The datasets analyzed during the study are available from the corresponding author on reasonable request.
